# Long-term Exposure to Ambient PM2.5 and Self-Reported Health: Evidence from Longitudinally-linked Census Data.

**DOI:** 10.23889/ijpds.v7i3.1809

**Published:** 2022-08-25

**Authors:** Neil Rowland, Duncan McVicar, Babak Jahanshahi, Mark McGovern, Dermot O'Reilly

**Affiliations:** 1 Queen's University Belfast; 2 Rutger's University

## Objectives

This paper estimates associations between long-term exposure to ambient particulate air pollution and 13 self-reported health outcomes including poor general health, chronic illness, experiencing long-running difficulties with breathing, and experiencing frequent periods of confusion or memory loss. It examines the extent to which these associations are explained by confounding factors.

## Approach

Longitudinally-linked Census data from the Northern Ireland Longitudinal Study linked to data on annual average particulate (PM2.5) concentrations at the 1km grid-square level over the period 2002-2010, exploiting complete residential histories, are used. The paper controls for potentially confounding factors at the neighbourhood, household and individual level, including for prior health, in a multivariate regression framework. Robustness to the presence of remaining unobserved confounders is assessed in two extensions, first by assuming selection on unobservables is proportional to selection on observables, and second through inclusion of an extensive set of fixed effects in the model.

## Results

There are strong statistical associations between long-term exposure to ambient particulate pollution and all 13 health outcomes measured by the 2011 Northern Ireland Census. Most of these estimated associations survive conditioning on an extensive set of observable controls. Of these, however, only two associations – with chronic illness and with long-running breathing difficulties, where we might expect the strongest causal effects – survive further analysis designed to elicit robustness to selection on unobservables. The estimated magnitudes of these remaining effects are non-trivial. For example, a 5 µg/m3 difference in particulate exposure averaged over 9 years has a similar magnitude effect on the probability of reporting long-running breathing difficulties as the difference between those aged in their 20s and those in their 40s.

## Conclusion

This study provides evidence of substantial effects of long-term exposure to ambient particulates on the probabilities of experiencing chronic illness and long-running breathing difficulties. These are qualitatively robust to both observed and unobserved confounders. Associations between particulate exposure and other health outcomes in the study are shown to reflect confounders.

